# Overall, sex-and race/ethnicity-specific prevalence of thyroid dysfunction in US adolescents aged 12–18 years

**DOI:** 10.3389/fpubh.2024.1366485

**Published:** 2024-06-20

**Authors:** Jianzhou Chen, Lingling Zhang, Xiaowen Zhang

**Affiliations:** ^1^Department of Cardiology, Affiliated Drum Tower Hospital, Nanjing University School of Medicine, Nanjing, China; ^2^Department of Pediatrics, Yugan People’s Hospital, Shangrao, China; ^3^Department of Endocrinology, Affiliated Drum Tower Hospital, Nanjing University School of Medicine, Nanjing, China

**Keywords:** thyroid dysfunction, prevalence, adolescents, NHANES, sex, race/ethnicity

## Abstract

**Background:**

Thyroid dysfunction significantly affects the health and development of adolescents. However, comprehensive studies on its prevalence and characteristics in US adolescents are lacking.

**Methods:**

We investigated the prevalence of thyroid dysfunction in US adolescents aged 12–18 years using data from the National Health and Nutrition Examination Survey (NHANES) 2001–2002 and 2007–2012 cycles. Thyroid dysfunction was assessed using serum thyroid-stimulating hormone (TSH) and free thyroxine (fT4) measurements. We analyzed the prevalence across demographic subgroups and identified associated risk factors.

**Results:**

The study included 2,182 participants, representing an estimated 12.97 million adolescents. The group had a weighted mean age of 15.1 ± 0.06 years, with males constituting 51.4%. Subclinical hyperthyroidism emerged as the most prevalent thyroid dysfunction, affecting 4.4% of the population. From 2001–2002 to 2011–2012, subclinical hyperthyroidism remained consistent at 4.99% vs. 5.13% in the overall cohort. Subclinical and overt hypothyroidism was found in 0.41 and 1.03% of adolescents respectively, and overt hyperthyroidism was rare (0.04%). The prevalence of thyroid peroxidase antibody (TPOAb) and thyroglobulin antibody (TgAb) positivity in the overall population were 5.8 and 9.8%, respectively. Positivity for TgAb was risk factors for hypothyroidism, while older age, female and Black Americans were risk factors for hyperthyroidism. Female adolescents and adolescents with an older age were more likely to be positive for TPOAb and TgAb, while Black and Mexican Americans had a lower risk of TPOAb and TgAb positivity.

**Conclusion:**

Subclinical hyperthyroidism was the most common form of thyroid dysfunction, and its prevalence remained stable from 2001–2002 to 2011–2012. Notable disparities in the prevalence of hyperthyroidism and antibody positivity were observed among different age, sex and racial/ethnic groups.

## Introduction

Thyroid hormone plays a critical role in early neurocognitive development, growth, and energy metabolism throughout childhood and adolescence. Thyroid dysfunction, including hyperthyroidism and hypothyroidism, is prevalent among adults and has been associated with increased morbidity and mortality related to cardiovascular disease (1). Additionally, hypothyroidism has been documented to correlate with an increased risk of various other conditions, including diabetes mellitus, obesity, metabolic syndrome, fatigue, and depression (2–5). This association holds true even among adolescents aged 12–18 years (6). Children with thyroid dysfunction, such as hyperthyroidism, require a period of surveillance or even prompt treatment, according to recent guidelines (7).

The national prevalence of thyroid dysfunction among adults in the has been extensively studied using data from the National Health and Nutrition Examination Survey (NHANES) conducted during various periods spanning 1988–1994, 1999–2002, and 2007–2012 (8, 9). Estimates from these surveys indicate that subclinical hypothyroidism affects approximately 4.3% and subclinical hyperthyroidism affects around 3.2% of the US adult population (9). The prevalence of thyroid dysfunction in children exhibits significant geographic variability (10, 11). For instance, notable differences exist in the prevalence of Graves’ disease between the United States and the United Kingdom (12, 13). However, there is limited research on the epidemiology of thyroid dysfunction among US adolescents using nationally representative data. A recent NHANES-based study estimated the prevalence of subclinical hypothyroidism among adolescents to be 2.0% (6), using an upper reference thyroid stimulating hormone (TSH) level of 4.5 mIU/L commonly employed in adult populations. Notably, studies comprehensively examining the prevalence of thyroid dysfunction applying age-adjusted normal reference ranges, are lacking. Furthermore, changes in dietary iodine intake, as evidenced by reduced urinary iodine concentration in children after 2001–2004, may have influenced the prevalence and characteristics of thyroid dysfunction in this demographic (14).

We aimed to estimate the overall prevalence of thyroid dysfunction, as well as the prevalence across key demographic subgroups, in a nationally representative cohort of US adolescents. We also aimed to identify risk factors contributing to thyroid dysfunction within this population.

## Methods

### Study design

The NHANES, initiated in 1999 and conducted biennially, are a series of cross-sectional surveys executed by the National Center for Health Statistics of the Centers for Disease Control and Prevention (15). NHANES systematically samples noninstitutionalized individuals across the US, providing a population-based perspective. Detailed descriptions of its study design and methodologies are well-documented elsewhere (16). In brief, the NHANES employs interviews, physical examinations, and laboratory analyses to evaluate the health of the non-institutionalized civilian in the US. The survey protocols have received approval from the National Center for Health Statistics Ethics Review Board, and informed consent was obtained from all participants.

Our study utilized data from NHANES spanning the years 2001 to 2002 and 2007 to 2012, which provided information on measurements of TSH and free thyroxine (fT4). Notably, data from the 2003 to 2006 cycles were omitted due to the absence of reported thyroid hormone data during that period. The study population consisted of participants aged 12–18 years, with exclusion criteria for missing data or pregnancy.

### Thyroid dysfunction

Thyroid dysfunction was evaluated through the measurement of serum TSH and fT4 concentrations. In the NHANES data sets, serum TSH from participants was measured with a two-site (sandwich) immunoenzymatic assay, fT4 was measured with a 2-step enzyme immunoassay, and TPOAb and TgAb titers were measured with a sequential two-step immunoenzymatic “sandwich” assay. We adhered to the established reference ranges for children and adolescents: 0.6–5.8 mU/L for TSH and 0.8–1.9 μg/dL for fT4 (17–21). In the absence of antithyroid drugs or thyroid hormone therapy, subclinical hypothyroidism was defined by elevated TSH (>5.8 mIU/L) with normal fT4 levels, and subclinical hyperthyroidism by reduced TSH (<0.6 mIU/L) with normal fT4 levels. Overt hypothyroidism was identified as a TSH level > 5.8 mIU/L with fT4 below the normal range, and overt hyperthyroidism was indicated by a TSH level < 0.6 mIU/L with fT4 above the reference range. Participants on thyroid hormone or antithyroid medication were classified as having overt hypothyroidism or hyperthyroidism, respectively.

A narrower TSH range of 0.4–4.5 mU/L was employed for sensitivity analysis (6). Thyroglobulin antibody (TgAb) or thyroid peroxidase antibody (TPOAb) positivity was defined as per NHANES standards (22). Due to the unavailability of thyroid disease history in participants under 20 years in NHANES, a disease-free population was delineated as those without antithyroid or thyroid hormone prescriptions.

### Statistical analysis

Statistical analyses were performed using Stata (version 16.0, StataCorp) and R (version 3.5.2, R Foundation), utilizing NHANES weights for nationally representative estimates (23). Categorical variables were presented as proportions (95% confidence interval [CI]), and continuous variables as means (95% CI). Prevalence differences between NHANES cycles were assessed for significance using an approximation of the Wald test, checking if one estimate’s central value fell within the other’s 95% CI (24). After confirming no temporal trends across cycles, data from all NHANES cycles were combined to improve precision and minimize sampling error, as per analytic guidelines (23).

Prevalence was calculated for both the overall and disease-free populations. The Taylor series (linearization) method estimated standard errors, while the Korn and Graubard method determined 95% CIs for prevalence (25). Subgroup analyses, based on sex, race/ethnicity, and TPOAb and TgAb status, examined prevalence within the overall population. Multivariate logistic regression models identified risk factors for thyroid dysfunction, with statistical significance set at a two-sided *p*-value of < 0.05.

## Results

### Participant characteristics

Our analysis encompassed 2,182 individuals, representing approximately 12.97 million US adolescents. The group had a weighted mean age of 15.1 ± 0.06 years, with males constituting 51.4%. Racial composition included 61.1% White people, 13.8% Black people, and 12.3% Mexican Americans. TPOAb and TgAb positivity were found in 5.7 and 9.7% of participants, respectively. Participants with hypothyroidism, compared to euthyroid individuals, were predominantly female, had a greater proportion of White people, and showed higher TPOAb and TgAb positivity rates. In contrast, hyperthyroid participants, also more likely to be female, had higher representation among Black people and Mexican Americans, though TPOAb or TgAb positivity rates did not differ significantly from the euthyroid group (Supplementary Table S1).

### Prevalence of thyroid dysfunction

In the overall population, the prevalence of hypothyroidism was 1.4% (95% CI, 0.6–2.7%), comprising 0.41% subclinical and 1.03% overt hypothyroidism (Table 1). In the disease-free population, these figures were 0.64% for overall hypothyroidism, 0.41% for subclinical and 0.23% for overt hypothyroidism.

**Table 1:** Prevalence of thyroid dysfunction among US adolescents aged 12–18 years.

	Hypothyroidism	Subclinical hypothyroidism	Overt hypothyroidism	Hyperthyroidism	Subclinical hyperthyroidism	Overt hyperthyroidism
Overall population	1.4 (0.6, 2.7)	0.4 (0.1, 1.1)	1.0 (0.4, 2.3)	4.5 (3.4, 5.7)	4.4 (3.3, 5.7)	0.04 (0, 0.3)
Disease-free population	0.6 (0.2, 1.4)	0.4 (0.1, 1.1)	0.2 (0.03, 0.8)	4.5 (3.4, 5.8)	4.4 (3.3, 5.7)	0.04 (0, 0.3)
**Sex**						
Female	2.1 (0.8, 4.5)	0.27 (0.02, 1.2)	1.8 (0.6, 4.3)	5.1 (3.5, 7.2)	5.0 (3.4, 7.1)	0.1 (0, 0.5)
Male	0.7 (0.2, 1.7)	0.55 (0.1, 1.7)	0.2 (0.04, 0.8)	3.8 (2.7, 5.2)	3.7 (2.6, 5.1)	0 (0, 0.3)
**Race and ethnicity**						
Non-Hispanic White	1.9 (0.7, 4.0)	0.4 (0.05, 1.6)	1.5 (0.5, 3.6)	4.2 (2.6, 6.4)	4.1 (2.5, 6.3)	0 (0, 0.6)
Non-Hispanic Black	0.7 (0.2, 1.8)	0.2 (0, 1.4)	0.5 (0.06, 1.6)	6.1 (4.2, 8.5)	6.1 (4.2, 8.5)	0 (0, 0.7)
Mexican American	0.9 (0.3, 2.2)	0.7 (0.2. 2.0)	0.2 (0.01, 1.0)	5.9 (4.0, 8.3)	5.6 (3.8, 7.9)	0.31 (0.01, 1.7)
Other	0.5 (0.04, 1.9)	0.2 (0, 1.5)	0.2 (0, 1.5)	2.7 (1.2, 5.2)	2.7 (1.2, 5.2)	0 (0, 1.0)
**TPOAb**						
Positive	7.2 (1.3, 21.1)	0.6 (0, 5.1)	7.2 (1.3, 21.1)	3.0 (0.5, 9.2)	3.0 (0.5, 9.2)	0.7 (0, 5.3)
Negative	0.7 (0.2, 1.7)	0.4 (0.1, 1.1)	0.7 (0.2, 1.7)	4.5 (3.4, 5.9)	4.5 (3.4, 5.9)	0 (0, 0.18)
**TgAb**						
Positive	5.4 (1.3, 14.2)	1.5 (0.1, 6.3)	5.4 (1.3, 14.2)	2.4 (0.6, 6.1)	2.4 (0.6, 6.1)	0.4 (0, 3.0)
Negative	0.6 (0.1, 1.6)	0.3 (0.05, 0.9)	0.6 (0.1, 1.6)	4.5 (3.4, 6.0)	4.5 (3.4, 6.0)	0 (0, 0.19)

TgAb, thyroglobulin antibody; TPOAb, thyroid peroxidase antibody.

**Figure 1:**
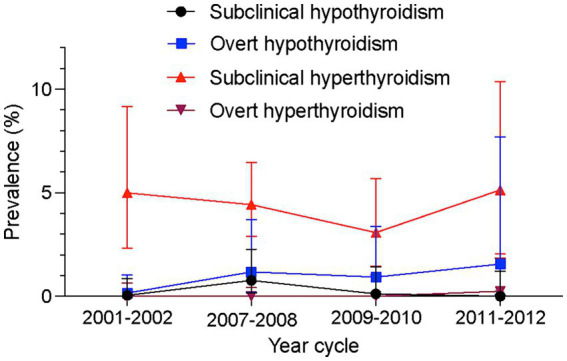
Trends in prevalence of thyroid dysfunction among US adolescents aged 12–18 years, 2001–2002 to 2011–2012.

For hyperthyroidism, the prevalence was 4.5% (95% CI, 3.4–5.7%), with 4.4% subclinical and 0.04% overt hyperthyroidism (Table 1). This prevalence was mirrored in the disease-free population. From 2001–2002 to 2011–2012, subclinical hyperthyroidism remained consistent at 4.99% vs. 5.13% in the overall cohort (Figure 1).

The proportion of TPOAb and TgAb positivity in the overall population were 5.8% (95% CI, 4.3–7.6%) and 9.8% (95% CI, 7.9–11.9%), respectively (Table 2). These rates were slightly lower in the disease-free population, at 5.4 and 9.3%, respectively (Table 2). All TPOAb-positive adolescents were also TgAb-positive, whereas 60.5% of those with TgAb positivity had TPOAb positivity (Table 2).

**Table 2:** Prevalence of antibody abnormality among US adolescents aged 12–18 years.

	TPOAb positive	TgAb positive
Overall population	5.8 (4.3, 7.6)	9.8 (7.9, 11.9)
Disease-free population	5.4 (4.0, 7.1)	9.3 (7.6, 11.3)
**Sex**		
Female	8.2 (5.8, 11.2)	12.2 (9.4, 15.5)
Male	3.2 (2.0, 4.7)	7.1 (5.2, 9.6)
**Race and ethnicity**		
Non-Hispanic White	7.4 (5.1, 10.3)	12.0 (9.3, 15.1)
Non-Hispanic Black	0.9 (0.3, 3.2)	3.4 (1.9, 5.6)
Mexican American	4.9 (3.2, 7.0)	8.0 (5.8, 10.6)
Other	3.9 (2.1, 6.8)	7.7 (4.5, 12.1)
**TPOAb**		
Positive	–	100 (96.0, 100)
Negative	–	39.8 (29.3, 52.8)
**TgAb**		
Positive	60.5 (50.4, 70.1)	–
Negative	0 (0, 0.2)	–

TgAb, thyroglobulin antibody; TPOAb, thyroid peroxidase antibody.

Using a TSH cutoff of 0.4–4.5 mU/L, the prevalence of subclinical and overt hypothyroidism was 1.7 and 0.92%, respectively, while subclinical and overt hyperthyroidism were 2.28 and 0.04%, respectively (Supplementary Figure S1).

### Risk factors for thyroid dysfunction

Adolescents testing positive for TgAb were at a sixfold increased risk of hypothyroidism compared to negative counterparts (OR 6.0, 95% CI 1.19–30.7, *p* = 0.031), but no significant differences were noted in hypothyroidism prevalence based on age, gender, race, ethnicity, or TPOAb status (Table 3).

**Table 3:** Factors associated with thyroid dysfunction and antibody abnormality among US adolescents aged 12–18 years.

	Hypothyroidism		Hyperthyroidism		TPOAb positive		TgAb positive	
	Odds ratio (95% CI)	*p* value	Odds ratio (95% CI)	*p* value	Odds ratio (95% CI)	*p* value	Odds ratio (95% CI)	*p* value
Age	1.1 (0.8, 1.5)	0.49	1.2 (1.0, 1.4)	0.04	1.2 (1.0, 1.4)	0.049	1.2 (1.0, 1.3)	0.02
**Sex**								
Male	Reference		Reference		Reference		Reference	
Female	2.1 (0.7, 6.6)	0.19	1.9 (1.3, 2.8)	0.002	2.7 (1.7, 4.3)	<0.001	1.8 (1.2, 2.6)	0.003
**Race and ethnicity**								
Non-Hispanic White	Reference		Reference		Reference		Reference	
Non-Hispanic Black	0.6 (0.2, 1.8)	0.34	1.8 (1.1, 3.0)	0.03	0.1 (0.04, 0.4)	<0.001	0.3 (0.2, 0.5)	<0.001
Mexican American	0.6 (0.2, 2.0)	0.72	1.6 (0.9, 2.8)	0.09	0.7 (0.4, 1.1)	0.14	0.7 (0.5, 0.96)	0.03
Other	0.3 (0.06, 1.5)	0.14	1.1 (0.6, 2.1)	0.78	0.5 (0.3, 0.99)	0.046	0.6 (0.4, 1.1)	0.09
**TPOAb**								
Negative	Reference		Reference					
Positive	1.1 (0.2, 7.9)	0.91	1.0 (0.2, 4.3)	0.96				
**TgAb**								
Negative	Reference		Reference					
Positive	6.0 (1.2, 30.7)	0.03	0.7 (0.2, 2.5)	0.56				

BMI, body mass index; TgAb, thyroglobulin antibody; TPOAb, thyroid peroxidase antibody.

Hyperthyroidism prevalence was significantly higher in adolescents as age grows (OR 1.17, 95% CI 1.01–1.35, *p* = 0.035), in female adolescents compared to males (OR 1.89, 95% CI 1.27–2.83, *p* = 0.002), and among Black people compared to White people (OR 1.78, 95% CI 1.07–2.99, *p* = 0.028). TPOAb or TgAb status did not significantly affect hyperthyroidism prevalence.

Female adolescents and adolescents with an older age were more likely to be positive for TPOAb and TgAb. Black and Mexican Americans had a lower risk of TPOAb and TgAb positivity than White Americans (Table 3).

## Discussion

Our study represents the first nationally representative investigation in the United States aimed at determining the prevalence of thyroid dysfunction among adolescents. Benefiting from a substantial participant pool, we showed that subclinical hyperthyroidism was the most common form of thyroid dysfunction, affecting 4.4% of the population, with 104 adolescents exhibiting this condition. Conversely, the prevalence of hypothyroid dysfunction was relatively low, impacting <1.0% of adolescents. Overt hyperthyroidism was identified as an rare occurrence among adolescents.

Our findings revealed a notable disparity in the prevalence of hyperthyroidism among different racial and ethnic groups. Non-Hispanic Black people exhibited approximately 1.8 times the likelihood of developing hyperthyroidism compared to non-Hispanic White people. However, no significant difference was observed in the prevalence of hypothyroidism among Black people, Mexican Americans, and White people. White people were more likely to test positive for TPOAb and TgAb, respectively, in comparison to Black people. It is worth noting that, to the best of our knowledge, no previous study has specifically addressed this issue of racial and ethnic differences in the prevalence of thyroid dysfunction in a nationally representative adolescent population in the US. In a population-based study conducted by McLeod et al., utilizing the NHANES database, it was demonstrated that Black people had a higher likelihood of having prevalent thyrotoxicosis than White people, which aligns with our findings. It is important to highlight that the McLeod study encompassed a broader age range, including adolescents and adults aged 12–49 years, whereas our study focused exclusively on adolescents (26).

In our analysis, we observed that female had a 1.9 times higher likelihood of developing hyperthyroidism compared to male. Furthermore, female were approximately 1.8–2.7 times more likely to test positive for TPOAb and TgAb. However, no significant difference in the risk of hypothyroidism was observed between female and male. It is worth noting that studies investigating sex differences in the risk of thyroid dysfunction are scarce in the existing literature, and none have been conducted on a national scale within the US. Interestingly, our findings align with a nationwide study conducted in France, which also reported a higher prevalence of Graves’ disease, the main form of hyperthyroidism in children and adolescents, among girls compared to boys. This study also revealed a significant interaction between age and sex, indicating that as age increases, the ratio of affected girls to boys also increases (27). Similarly, in our study, we observed a higher risk of hyperthyroidism as age advanced among adolescents. However, no such association was found for hypothyroidism.

An interesting finding from our study was the higher prevalence of TgAb positivity compared to TPOAb positivity among adolescents, and that all adolescents who tested positive for TPOAb were also positive for TgAb. In a NHANES III analysis, the overall population showed a prevalence of 13.0 and 11.5% for TPOAb and TgAb positivity, respectively (28). However, among individuals with positive TPOAb, 54.5% also had positive TgAb. These findings highlight distinct profiles of TPOAb and TgAb between adolescents and adults. It is evident that both TPOAb and TgAb serve as indicators of thyroid autoimmunity, albeit with different specific applications in clinical practice. The differences observed in their prevalence and co-occurrence suggest the need for future studies to unravel the underlying mechanisms driving these distinctions.

Considering the relatively low incidence of thyroid dysfunction in adolescents, it may be justified to forego routine thyroid screening in the general pediatric population, instead focusing on specific conditions and disease contexts. While population-based studies have documented the association between subclinical hypothyroidism and cardiometabolic risk factors in young individuals (6), such a relationship with hyperthyroidism has not been established. Future longitudinal investigations are necessary to ascertain whether subclinical hyperthyroidism may elevate the risk for these cardiometabolic factors, potentially leading to adverse cardiometabolic outcomes.

This study’s limitations encompass the limited number of cases for overt hyperthyroidism, lack of follow-up of thyroid function in the included individuals from the NHANES datasets, lack of information regarding puberty, lack of information on supplement intake which can affect thyroid function, absence of information on the etiology of thyroid dysfunction, along with the absence of geographic data and data from the most recent decade.

## Conclusion

In conclusion, our study provides crucial insights into the prevalence of thyroid dysfunction among U.S. adolescents, highlighting subclinical hyperthyroidism as the most common form. Significant racial and gender disparities in hyperthyroidism prevalence were observed, with non-Hispanic Black people and females showing higher risks. These findings underscore the need for ongoing research to better understand and manage thyroid dysfunction in adolescents, considering evolving dietary and environmental factors.

## Data availability statement

The original contributions presented in the study are included in the article/Supplementary material, further inquiries can be directed to the corresponding author.

## Ethics statement

The studies involving humans were approved by National Center for Health Statistics Ethics Review Board. The studies were conducted in accordance with the local legislation and institutional requirements. Written informed consent for participation in this study was provided by the participants’ legal guardians/next of kin.

## Author contributions

JC: Investigation, Writing – original draft, Formal analysis. LZ: Conceptualization, Writing – review & editing, Investigation. XZ: Formal analysis, Writing – original draft, Writing – review & editing, Conceptualization, Funding acquisition, Methodology, Supervision.
