# Supplementary material for: Overall, sex-and race/ethnicity-specific prevalence of thyroid dysfunction in US adolescents aged 12–18 years
Source: Front Public Health. 2024 Jun 20;12:1366485. doi: 10.3389/fpubh.2024.1366485 (PMC11222593; doi:10.3389/fpubh.2024.1366485)
Supplement: Supplementary file 1 [file Data_Sheet_1.DOCX]

**SUPPLEMENTARY MATERIALS**

**Overall, Sex- and Race/ethnicity-Specific Prevalence of Thyroid Dysfunction in US Adolescents Aged 12-18 Years**

**Table S1. Characteristics of US adolescents aged 12 to 18 years from NHANES based on thyroid function status.**

|  | **Overall** | **Hypothyroidism** | **Hyperthyroidism** | **Euthyroid** |
| --- | --- | --- | --- | --- |
| **Age, years** | 15.1 (15.0, 15.2) | 15.7 (13.5, 18.0) | 15.4 (14.9, 16.0) | 15.0 (14.9, 15.1) |
| **Sex** |  |  |  |  |
| Female | 51.4 (48.9, 53.9) | 74.6 (20, 97.2) | 59.3 (46.8, 70.6) | 50.2 (47.9, 52.5) |
| Male | 48.6 (46.1, 51.1) | 25.3 (2.8, 80.0) | 40.7 (29.4, 53.2) | 49.8 (47.5, 52.1) |
| **Race and ethnicity** |  |  |  |  |
| Non-Hispanic White | 61.1 (56.5, 65.5) | 81.1 (39.4, 96.7) | 56.7 (41.9, 70.5) | 61.4 (56.8, 65.8) |
| Non-Hispanic Black | 13.8 (11.5, 16.4) | 6.6 (0.9, 35.8) | 18.8 (12.0, 28.3) | 13.5 (11.2, 16.1) |
| Mexican American | 12.3 (10.0, 15.1) | 8.0 (0.9, 46.5) | 16.6 (10.2, 25.7) | 12.2 (9.9, 14.8) |
| Other | 12.8 (9.8, 16.6) | 4.2 (0.2, 51.8) | 7.9 (3.4, 17.2) | 13.0 (9.9, 16.8) |
| **TPOAb positivity** | 5.7 (4.3, 7.6) | 32.0 (2.6, 89.1) | 4.8 (1.6, 13.3) | 5.4 (4.1, 7.3) |
| **TgAb positivity** | 9.5 (7.8, 11.6) | 47.2 (6.5, 92.0) | 6.1 (2.5, 14.5) | 9.1 (7.4, 11.3) |

Categorical data are presented as percentage (95% CI).

TgAb, thyroglobulin antibody; TPOAb, thyroid peroxidase antibody.


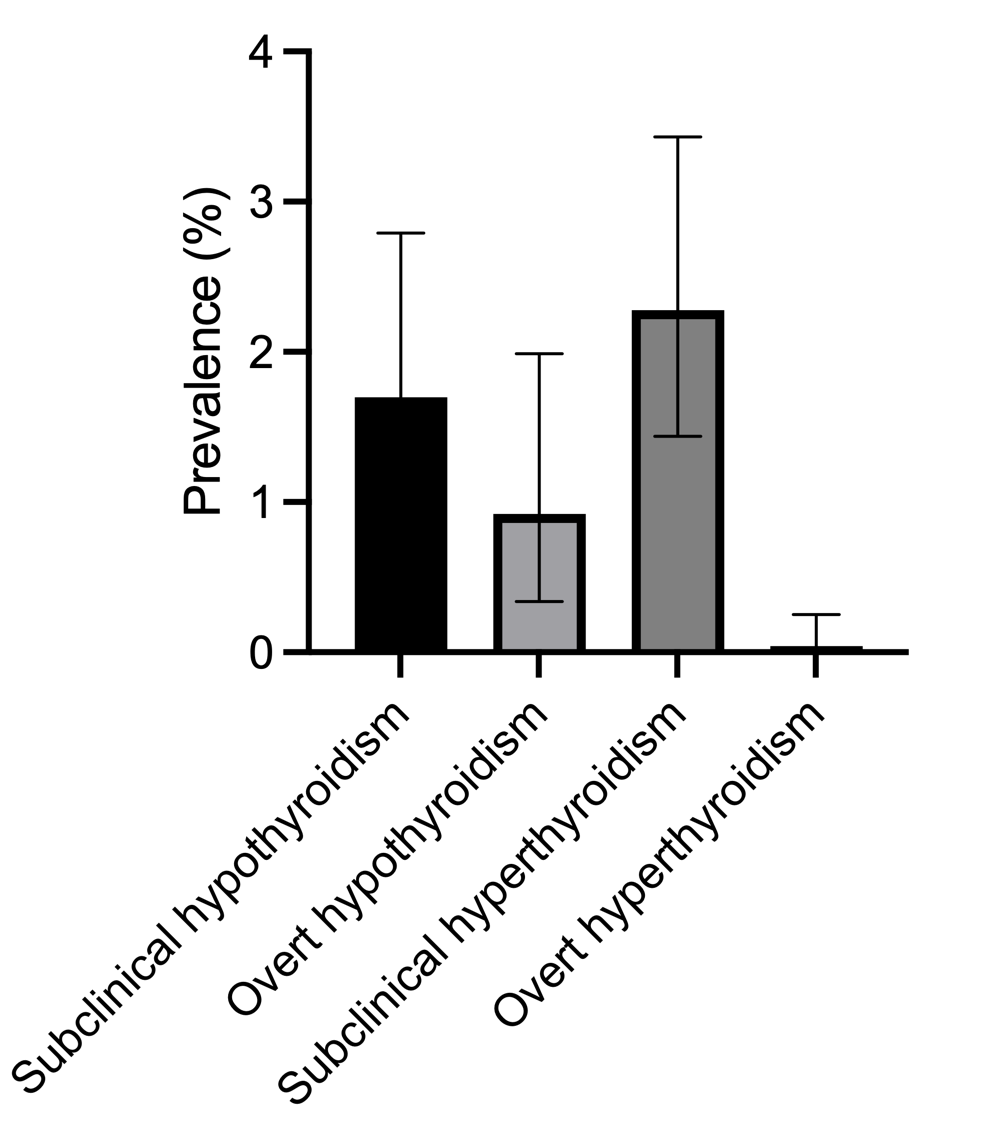


**Figure S1. Prevalence of thyroid dysfunction among US adolescent aged 12-18 years in the overall population using a TSH cutoff of 0.4-4.5 mU/L.**
